# A Novel Hepadnavirus is Associated with Chronic Hepatitis and Hepatocellular Carcinoma in Cats

**DOI:** 10.3390/v11100969

**Published:** 2019-10-21

**Authors:** Patricia A. Pesavento, Kenneth Jackson, Timothy Scase, Tiffany Tse, Bronte Hampson, John S. Munday, Vanessa R. Barrs, Julia A. Beatty

**Affiliations:** 1 Department of Pathology Microbiology and Immunology, UC Davis, Davis, CA 95616, USA; papesavento@ucdavis.edu (P.A.P.); kajackson@ucdavis.edu (K.J.); tytse@ucdavis.edu (T.T.); 2 Bridge Pathology, Bristol BS7 0BJ, UK; timscase2.0@gmail.com; 3 Faculty of Science, Sydney School of Veterinary Science, University of Sydney, Camperdown, NSW 2006, Australia; bham3841@uni.sydney.edu.au (B.H.); vanessa.barrs@sydney.edu.au (V.R.B.); 4 School of Veterinary Science, Massey University, Palmerston North 4410, New Zealand; J.Munday@massey.ac.nz

**Keywords:** feline, cancer, viral, cat, HBV, HCC, disease, oncogenesis, pathology, *Orthohepadnavirus*, hepatology

## Abstract

In 2015, over 850,000 people died from chronic hepatitis and hepatocellular carcinoma (HCC) caused by hepatitis B virus (HBV). A novel hepatitis B-like virus has recently been identified in domestic cats. The pathogenic potential of domestic cat hepadnavirus (DCH), for which 6.5% to 10.8% of pet cats are viremic, is unknown. We evaluated stored formalin-fixed, paraffin-embedded biopsies of diseased and normal feline liver for the presence of DCH using PCR and in situ hybridization (ISH). DCH was detected in 43% (6/14) of chronic hepatitis cases and 28% (8/29) of HCCs, whereas cholangitis (*n* = 6), biliary carcinoma (*n* = 18) and normal liver (*n* = 15) all tested negative for DCH. Furthermore, in DCH-associated cases, the histologic features of inflammation and neoplasia, and the viral distribution on ISH were strikingly similar to those seen with HBV-associated disease. Several histological features common in human HBV-associated hepatitis, including piecemeal necrosis and apoptotic bodies, were identified in DCH-positive cases of chronic hepatitis. In two cases of HCC examined, the proliferation index in regions that were ISH-positive was higher than in ISH-negative regions. The intracellular distribution of virus in both hepatitis and HCC demonstrated that viral nucleic acid is present in both nuclear and cytoplasmic forms. Collectively, these findings demonstrate a compelling association between DCH and some cases of chronic hepatitis and hepatocellular carcinoma in the cat that mirrors features of HBV-associated hepatopathies. Future investigations of viral epidemiology and natural history are needed to establish the impact of DCH on feline health.

## 1. Introduction

The Hepadnaviridae is a family of partially double-stranded DNA viruses exhibiting a narrow host-range and strong hepatotropism. Over 257 million people are chronically infected with the type species, Hepatitis B virus (HBV) [1]. Chronic HBV infection can trigger immune-mediated chronic hepatitis that is characterized by necrosis, regeneration, and fibrosis, progressing to cirrhosis and hepatocellular carcinoma (HCC) [2]. The complex pathogenesis of HBV-associated hepatopathies, and the interplay between the virus and diverse risk factors, remain incompletely understood [3]. Despite an effective HBV vaccine, significant challenges to global HBV control remain.

In 2018, a novel, naturally occurring hepadnavirus, domestic cat hepadnavirus (DCH), was discovered [4]. DCH infection appears to be common in cats with viremia, detected in 6.5% and 10.8% of pet cats in Australia and Italy respectively [4,5]. The potential of DCH to contribute to feline liver disease, if any, is not yet known. Elevations in viral load and serum alanine aminotransferase (ALT), biomarkers of HBV progression, in some DCH-infected cats provide indirect support for a possible role for DCH in feline liver disease [5]. Chronic hepatitis and HCC, potential sequelae of HBV infection in humans, also occur in domestic cats, but they have been considered to be idiopathic in this species [6]. In this international, multicenter study, we used PCR and in situ hybridization (ISH) to identify hepatotropism, the cellular targets, and the distribution of DCH in normal and diseased feline liver samples.

## 2. Materials and Methods

### 2.1. Case Selection and Tissues

Formalin-fixed, paraffin-embedded (FFPE) samples of feline liver were obtained from 4 institutions in 4 countries (University of California at Davis, USA; University of Sydney, Australia; Massey University, New Zealand; Bridge Pathology, Bristol, UK). Samples were obtained either by routine biopsy or necropsy, and all were collected by owner consent. Cases that represented a spectrum of commonly diagnosed liver diseases in the cat were selected. Study inclusion required the availability of histologic sections that allowed confirmation of microscopic features consistent with the diagnosis. Blinded histology review was carried out by a single pathologist (PP) for the final classification. Cases of chronic (interface-type) hepatitis were selected using the current internationally accepted definition [7]. Cases identified as cholangitis had inflammation clearly centered on or around biliary ductules without impact, or with only regional dropout, of hepatocytes. In cases categorized as HCC, there was loss of lobular architecture, thickening of hepatocellular cords, lobular compression and/or invasion, and mild to marked cellular atypia. Normal liver controls were defined by the absence of any histological features of disease and a normal serum ALT concentration. Cases and controls were further selected based on the quality (lack of autolytic change, <1 week in 10% buffered formalin) and quantity of the sampled liver tissue. Only samples with adequate preservation of nucleic acid, based on amplification of GAPDH in DNA, were included.

### 2.2. DNA Extraction

Sections, 25 µM thick, of FFPE feline liver were dewaxed with 1 mL of xylene three times and washed with 1 mL of 100% ethanol, 1 mL of 90% ethanol, and 1 mL of 70% ethanol. The tissue was digested overnight in 180 µL of Qiagen ATL buffer and 20 µL proteinase K solution. DNA was purified using the DNeasy Blood and Tissue kit (Qiagen, Hilden, Germany) according to the manufacturer’s instructions and eluted using 100 μL of DNA elution buffer and stored at −20 °C.

### 2.3. Conventional PCR Assays

**Figure 1:**
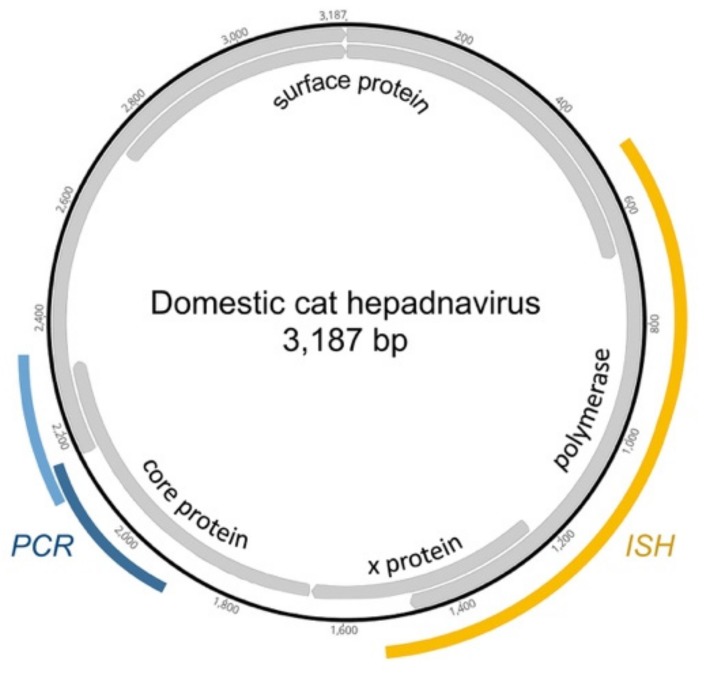
Domestic cat hepadnavirus genome. There are four ORFs. PCR probes used for this study (blue) are partially overlapping and span the junction between the core and polymerase genes. The core and polymerase genes in the ortholog hepatitis B virus (HBV) are required for replication. The ISH probe V-FeHepadnavirus (yellow) is a 20 ZZ paired probe set hybridizing to target DNA or transcript of the X and polymerase genes.

Two conventional PCR (cPCR) assays were used to screen DNA extracted from FFPE liver for presence of DCH. Primer set 1 (Hgap-F/Hgap-R) [4] amplifies a 230 bp region of the viral genome, and primer set 2 (FeHep.2116F (GCACCTGGATTCGCACAC)/FeHep.2371R (CCTTGAGGGAGTAAAGCCCTG)) amplifies a 256 bp region (Figure 1). The 25 μL reactions contained 12.5 μL HotStarTaq Plus Master Mix (Qiagen), 0.5 μM forward primer, 0.5 μM reverse primer, 2.5 μL dye, and 100–200 ng of purified DNA. Cycling conditions were an initial activation step of 95 °C for 5 min, followed by 40 cycles of 94 °C for 30 s, 55 °C for 30 s (52 °C for primer set 2), and 72 °C for 30 s, with a final elongation step at 72 °C for 10 min. Amplicons were evaluated by 1.5% agarose gel electrophoresis. The identity of bands of the correct size was confirmed by cloning, using the TOPO TA Cloning kit (Invitrogen, Thermo Fisher Scientific, Waltham, MA, USA), Sanger sequencing (DNA sequencing facility, UC Davis).

### 2.4. In Situ Hybridization

We designed an antisense probe, V-FeHepadnavirus, (Advanced Cell Diagnostics, Inc., Hayward, CA, USA) targeting region 604–1477 of DCH, Genbank accession number MH3079301 (Figure 1) [4]. Cases that tested positive for DCH by cPCR, and negative control tissues, were progressed to ISH. Colorimetric ISH was performed manually on 5 μm sections of FFPE tissue on Superfrost Plus slides (Fisher Scientific, Pittsburgh, PA, USA) using the RNAscope 2.5 Red assay kit (Advanced Cell Diagnostics, Inc.). Each section was pretreated with heat and protease prior to probe hybridization for 2 h at 40 °C.

For signal validation, negative control probes were used to probe serial sections, including a probe designed to detect dihydrodipicolinate reductase (DapB) of *Escherichia coli* (all cases). Uninfected, histologically normal feline liver tissue served as a negative tissue control (*n* = 3). Slides were counterstained with hematoxylin and mounted with EcoMount (Biocare Medical, Concord, CA). Slides were digitized using an Olympus VS120 scanner and a 40× objective with bright-field illumination.

### 2.5. Immunohistochemistry

For DCH-associated HCC, the proliferation index was determined in two representative cases by immunohistochemistry using an anti-Ki67 antibody, MIB1, a mouse monoclonal antibody (Agilent, DAKO, Santa Clara, CA, USA). Antigen retrieval was steam for 30 min. The proliferation index was calculated in 10 high-power fields, each containing viral-positive and viral-negative regions, and presented as the percentage of Ki67-positive cells relative to the total number of hepatocytes.

## 3. Results

### 3.1. Cases and Controls

In total, biopsies from 71 individual cats with 80 lesions were included; chronic hepatitis (*n* = 14), cholangitis (*n* = 6), HCC (*n* = 29), biliary carcinoma (*n* = 18), nodular hyperplasia (*n* = 8), multilocular biliary cysts (*n* = 4), and toxic (amanitin) hepatopathy (*n* = 1). Nodular hyperplasia and cysts were concurrent, in some cases, with hepatitis or found in otherwise normal livers. Fifteen normal livers samples served as controls.

### 3.2. Detection of DCH in Feline Liver Biopsies

Using PCR, DCH DNA was amplified from 6 of 14 (43%) cases of chronic hepatitis and 8 of 29 (28%) HCCs. DCH DNA was not amplified from any sample of biliary carcinoma, cholangitis, nodular hyperplasia, multilocular biliary cysts, toxic hepatopathy, or from normal liver. Two of 6 DCH-positive chronic hepatitis cases were positive by ISH, whereas all PCR-positive HCCs were also positive for DCH by ISH (Table 1).

### 3.3. Character of DCH-Associated Lesions by Traditional Pathology and ISH

#### 3.3.1. Chronic Hepatitis

**Figure 2:**
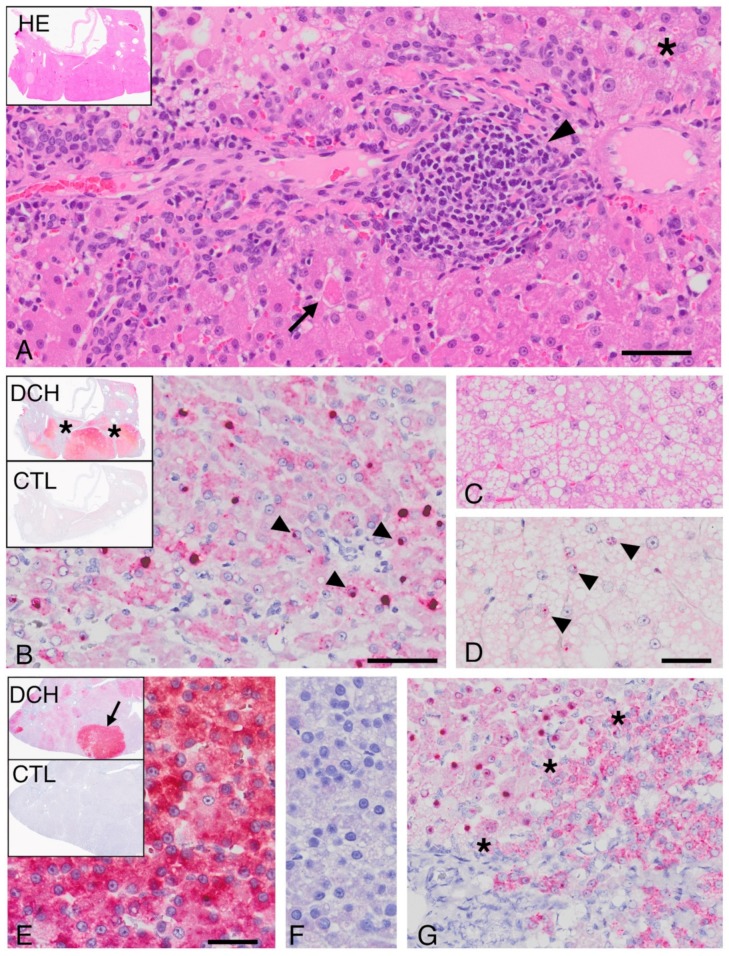
DCH associated chronic hepatitis (**A**–**D**) and DCH-associated hepatocellular carcinoma (**E**–**G**). (**A**) Lymphocytes are present clustered near portal tracts (arrowhead) and within sinusoids. Scattered, variably sized groups of hepatocytes are vacuolated (asterisk). An individual hepatocyte (arrow) is hypereosinophilic and individualized (necrosis). Inset, overview, medial lobe. Hematoxylin and eosin, Bar = 50 microns. (**B**) Chronic hepatitis, in situ hybridization. Inset, in this low magnification overview there is DCH probe hybridization (red) in the vast majority of the section. Collapsed lobules and fibrous tracts fail to hybridize (asterisks). DCH ISH demonstrates a combination of nuclear (arrowheads) and cytoplasmic hybridization, ISH, Bar = 50 microns. (**C**) There are scattered regions of vacuolar degeneration. Hematoxylin and eosin. (**D**) In a serial section, probe hybridization is limited to eccentric “dots” within nuclei. ISH. Bar = 50 microns. (**E**) DCH-associated HCC using an ISH probe for DCH at low magnification (inset, top) demonstrates the nodular HCC region with intense cytoplasmic hybridization (arrow) that is viewed by higher magnification in the larger panel. Bar = 50 microns. Using a control probe, no hybridization is present on a serial section of the same nodule (inset, bottom) and (**F**). (**G**) In other regions of the tumor there is more variability in probe hybridization. The asterisks define the border of hepatocytes with both nuclear and cytoplasmic hybridization (left) directly abutting hepatocytes where detectable hybridization was limited to the cytoplasm (right).

Chronic hepatitis cases positive by PCR for DCH displayed lymphocytic periportal inflammation (Figure 2A), with some regions including inflammation at the portal–lobular interface characteristic of “piecemeal necrosis”. Lymphocytes and plasma cells were also scattered within sinusoidal spaces where they sometimes clustered. Neutrophils were rare, and when present they were associated with regions of individual cells, or interface necrosis. Fibrosis irregularly spanned portal regions and dissected briefly into regional sinusoids. All DCH-associated chronic hepatitis cases, but also several of the DCH-negative cases, had foci of mild to marked hepatocellular dysplasia and nodular to poorly demarcated regions of vacuolated hepatocytes (Figure 2C).

The pattern of hybridization in cases of DCH-associated chronic hepatitis was patchy (Figure 2B). Regions that were diffusely and strongly positive abutted other areas that had scant or no hybridization. The regional variation usually corresponded to nodules (Figure 2, inset. top). The pattern of hybridization within individual cells also varied. Most hepatocytes that were positive for DCH on probe hybridization had a combination of nuclear and cytoplasmic signals (Figure 2B), but in some individual hepatocytes probe hybridization was limited to an eccentric single “dot” within the nucleus (Figure 2D).

#### 3.3.2. Hepatocellular Carcinoma

Neoplasms characterized as HCC were invasive, demonstrated loss of lobular architecture, and in most neoplastic regions sinusoids were either inapparent (solid) or hepatocellular cords were deeply piled. There was variable cellular atypia, and most HCC cases contained foci of confluent necrosis. Vacuolar degeneration was seen in DCH-positive HCCs and interpreted to be a combination of fatty change and glycogen accumulation, based on periodic acid–Schiff stain.

**Figure 3:**
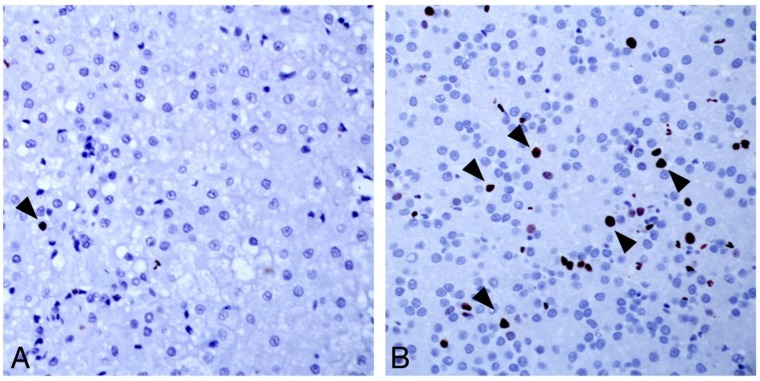
Ki67 immunohistochemistry of a DCH-negative region of HCC (**A**), and a DCH-positive region of HCC (**B**). Virus positive regions were defined by ISH. Arrowheads in both (**A**) and (**B**) denote hepatocyte nuclei expressing Ki67. Cell cycling is higher in regions of the tumor that are positive for DCH.

As was true for the cases of DCH-associated hepatitis, there were scattered to clustered cells in all HCCs where hybridization was cytoplasmic (Figure 2E), but in other regions there was nuclear localization. The variability in detection was striking (Figure 2E, inset. top). Entire nodules of neoplastic cells could be diffusely, intensively positive and sometimes abutted other nodules with less, or entirely absent, probe hybridization. Nuclear hybridization was either diffuse or restricted to an eccentric “dot” of signal at the periphery of the nucleus or filling the nucleus (Figure 2G). The variability in virus detection allowed an estimation of proliferation index in an individual case. Regions of DCH probe hybridization by ISH had a higher proliferation index (10–20 per HPF) than non-viral-associated regions (1–2 per HPF, Figure 3). HE = haematoxylin and eosin; DCH = ISH using probe for domestic cat hepadnavirus nucleic acid; CTL = control ISH probe.

### 3.4. DCH-Negative Lesions

Normal liver, biliary carcinomas, multilocular biliary cysts, and toxic hepatopathy were all negative for DCH by ISH.

## 4. Discussion

Hepadnaviruses that naturally infect primates, bats, and rodents cause liver diseases including chronic hepatitis and HCC, but the pathogenesis of these hepatopathies remains incompletely understood [8,9]. Here, for the first time, the feline hepadnavirus is demonstrated in association with lesions that mirror pathologies caused by HBV. Further, in DCH-associated cases, the histologic features of inflammation and neoplasia and the viral distribution were strikingly similar to those seen with HBV-associated disease.

Several microscopic features that are consistent with, but not pathognomonic for, human HBV-associated hepatitis, including “piecemeal” necrosis, apoptotic bodies, and sinusoidal inflammation [10], were identified in combination in cases of DCH-associated chronic hepatitis but not in DCH-negative hepatitis. Similarly, the character of DCH-associated HCC shared features with hepatopathies caused by HBV and/or woodchuck hepatitis virus, including regions of vacuolar change and individual hepatocellular necrosis, although individual histologic features of HCC did not distinguish DCH-positive from DCH negative cases. Greater hepatocyte proliferation observed in DCH-associated areas of HCC compared with virus negative regions is worthy of note since hepatocyte proliferation driven by the host immune response contributes to hepatocyte transformation in HBV-associated HCC [11]. The consistency of this finding should now be tested in a larger number of DCH-associated HCCs. HBV-associated HCC usually arises from a background of chronic inflammation and cirrhosis [12]. While the histologic character of DCH- and HBV-associated chronic hepatitis are similar, prospective studies would be needed to establish progression of disease from inflammation to neoplasia.

During replication, hepadnaviruses produce intranuclear DNA forms, including persistent, covalently closed circular DNA and integrated virus, as well as multiple cytoplasmic mRNAs [13]. The antisense ISH probe used in this study is designed to detect RNA, but in double-stranded DNA viruses, the probe will also detect the sense strand of genomic DNA. The pattern of hybridization revealed in DCH-positive tissues was regionally mixed in both intensity and in intracellular distribution (cytoplasmic, nuclear, or both). This pattern is consistent with the complex lifecycle of HBV and other hepadnaviruses [13]. Targeted, gene-specific ISH probes could assist in further elaborating the DCH lifecycle.

The ISH assay for DCH detection developed and validated here expands the options available for virus detection. While both PCR and ISH can detect DCH infection, a negative result for either test does not rule out infection. ISH is more sensitive than PCR in that it can detect single DCH-infected cells, but only a small number of cells are sampled for ISH, which may not be representative of all pathologic processes in the liver. This may explain why only two of six PCR-positive chronic hepatitis cases were positive for DCH on ISH, in contrast to all PCR-positive HCCs. If DCH-infected cells are less frequent in hepatitis than HCC, these cells may not be included in the biopsy. Alternatively, the PCR result may reflect viremia rather than infection of hepatocytes in the four ISH-negative hepatitis cases.

Collectively, the results of this molecular and morphologic study demonstrate a compelling association between DCH and some cases of chronic hepatitis and HCC. The similarity between the presence and distribution of virus in these lesions in the cat, and that in hepatopathies in human and woodchuck that are caused by HBV and WHV respectively, allows speculation that DCH may be one cause of chronic hepatitis and HCC in the cat. However, there are other explanations for our findings. Detection of virus within these lesions may be a coincidence, or a consequence of the disease, perhaps because of local upregulation of virus replication.

The potential impact of DCH on feline health will be of great interest to the global veterinary community because of the popularity of cats as human companion animals [14]. Chronic hepatitis is apparently uncommon in cats, being reported to occur at a frequency of 2.4% of all feline hepatic biopsies [6]. Primary hepatic neoplasia is estimated to account for 1% to 2.9% of all cancers [15,16]. Biliary carcinomas and HCC have been reported at a frequency of 17% and 27% of all feline hepatic neoplasms [6]. In our collections from the USA and UK over the past 10 years, HCC was the most common hepatic primary epithelial cancer (HCC = 71/132, 54%). On the other hand, in the two small studies undertaken to date, DCH viremia was detected in over 10% of cats depending on the population tested [4,5]. It is possible that liver disease is underdiagnosed in cats because veterinary diagnostic investigation is often limited, and biopsies, particularly needle biopsies, may not be representative of the pathologic processes throughout the liver. Whether DCH infection is apathogenic or associated with subclinical or clinical disease in cats remains to be determined. The prospective clinical investigation of cats with DCH viremia will assist in understanding the impact of DCH on feline health. If DCH is identified as a feline pathogen, then the reverse-translational potential of HBV treatments and prophylaxis could be investigated for cats.

## Figures and Tables

**Table 1:** Detection of domestic cat hepadnavirus (DCH) in feline liver by PCR and ISH.

Histopathological Diagnosis	Number of Cases	DCH Positive	
		cPCR	ISH
Hepatocellular carcinoma	29	8/29	8/8
Biliary carcinoma	18	0/18	0/11
Hepatitis	14	6/14	2/6
Cholangitis	6	0/6	0/5
Other ^1^	13	0/4	0/3
Normal liver ^2^	15	0/15	0/3

^1^ Nodular hyperplasia, multilocular biliary cysts, and toxic hepatopathy. Cysts and regions of nodular hyperplasia often occurred concurrently in cases of hepatitis and in otherwise normal livers. ^2^ Histologically normal and normal serum alanine aminotransferase.

## References

[1] World Health Organisation (2017). Global Hepatitis Report 2017.

[2] Guidotti L.G., Isogawa M., Chisari F.V. (2015). Host-virus interactions in hepatitis B virus infection. Curr. Opin. Immunol..

[3] Fattovich G., Bortolotti F., Donato F. (2008). Natural history of chronic hepatitis B: Special emphasis on disease progression and prognostic factors. J. Hepatol..

[4] Aghazadeh M., Shi M., Barrs V.R., McLuckie A.J., Lindsay S.A., Jameson B., Hampson B., Holmes E.C., Beatty J.A. (2018). A Novel Hepadnavirus Identified in an Immunocompromised Domestic Cat in Australia. Viruses.

[5] Lanave G., Capozza P., Diakoudi G., Catella C., Catucci L., Ghergo P., Stasi F., Barrs V., Beatty J., DeCaro N. (2019). Identification of hepadnavirus in the sera of cats. Sci. Rep..

[6] Bayton W.A., Westgarth C., Scase T., Price D.J., Bexfield N.H. (2018). Histopathological frequency of feline hepatobiliary disease in the UK. J. Small Anim. Pract..

[7] Van den Ingh T.S.G., Van Winkle T., Cullen J.M., Charles J.A., Desmet V.J., Rothuizen J., Bunch S.E., Charles J.A., Cullen J.M., Desmet V.J., Szatmári V., Twedt D.C., van den Ingh T.S., Van Winkle T., Washabau R.J. (2006). Morphological Classification of Parenchymal Disorders of the Canine and Feline Liver: 2. Hepatocellular Death, Hepatitis and Cirrhosis. WSAVA Standards for Clinical and Histological Diagnosis of Canine and Feline Liver Diseases.

[8] Glebe D., Urban S. (2007). Viral and cellular determinants involved in hepadnaviral entry. World J. Gastroenterol..

[9] Drexler J.F., Geipel A., König A., Corman V.M., Van Riel D., Leijten L.M., Bremer C.M., Rasche A., Cottontail V.M., Maganga G.D. (2013). Bats carry pathogenic hepadnaviruses antigenically related to hepatitis B virus and capable of infecting human hepatocytes. Proc. Natl. Acad. Sci. USA.

[10] Desmet V.J., Gerber M., Hoofnagle J.H., Manns M., Scheuer P.J. (1994). Classification of chronic hepatitis: Diagnosis, grading and staging. Hepatology.

[11] Dandri M., Petersen J. (2016). Mechanism of Hepatitis B Virus Persistence in Hepatocytes and Its Carcinogenic Potential. Clin. Infect. Dis..

[12] Yang J.D., Kim W.R., Coelho R., Mettler T.A., Benson J.T., Sanderson S.O., Therneau T.M., Kim B., Roberts L.R. (2011). Cirrhosis Is Present in Most Patients With Hepatitis B and Hepatocellular Carcinoma. Clin. Gastroenterol. Hepatol..

[13] Tang L.S.Y., Covert E., Wilson E., Kottilil S. (2018). Chronic Hepatitis B Infection A Review. J. Am. Med. Assoc..

[14] American Veterinary Medical Association (2018). AVMA Pet Ownership and Demographics Sourcebook (2017–2018).

[15] Hammer A.S., Sikkema D.A. (1995). Hepatic Neoplasia in the Dog and Cat. Veter. Clin. N. Am. Small Anim. Pract..

[16] Cullen J.M. (2016). Tumors of the Liver and Gallbladder. Tumors in Domestic Animals.

